# Polymer Dynamics: Bulk and Nanoconfined Polymers

**DOI:** 10.3390/polym14071271

**Published:** 2022-03-22

**Authors:** Takashi Sasaki

**Affiliations:** Department of Materials Science and Engineering, University of Fukui, Fukui 910-8507, Japan; sasakita@u-fukui.ac.jp

The dynamics in polymeric systems affect various important properties including mechanical and thermal behaviors, and extensive studies in this field have been executed not only academically but also practically. The features of molecular motions of chain molecules in an amorphous state are complicated because of the dynamical cooperativity and connectivity of the segments. They are significantly affected by the chemistry of the polymer, the molecular orientation, crystallinity, surfaces and/or interfaces, and the size scale of confinement. In particular, it is well known that anomalous dynamics of polymer chains in nanoconfined systems such as ultrathin films have been attracting a great deal of attention in the polymer physics community. This Special Issue, entitled “Polymer Dynamics: Bulk and Nanoconfined Polymers” consists of research papers that concern a variety of topics in polymer dynamics. The issue has ultimately covered a wider range than was originally expected, a range which includes studies of the fundamentals of polymer dynamics, interfacial phenomena, behaviors of polyelectrolytes, swelling, crystallization, and the processes of polymer synthesis.

As a fundamental approach to understanding the dynamics in supercooled liquids including polymeric materials, a dynamically correlated network (DCN) model was proposed [1]. The DCN was assumed to consist of sequentially connected segments, and the size distribution and geometry of the cooperative cluster with respect to temperature were investigated via Monte Carlo simulations. It was revealed that the temperature dependence of the segmental relaxation time for various glass-forming liquids can be well described by the DCN model. Wu et al. proposed an original model for a system of mechanically interlocked molecules such as rotaxanes [2]. The sliding dynamics of rings along the two axial chain molecules (symmetric or asymmetric) were investigated via molecular dynamics simulations, where the rings are linked by a linear chain. It was revealed that the sliding dynamics depend on the bending energy of the axial chains and the distance between them. Moreover, it was suggested that the conformational entropy of the chain that connects the rings also plays an important role.

Two articles in this Special Issue are related to mechanical and rheological properties. Microinjection molding techniques that are used to fabricate polymer microparts are becoming important in various applications. The shear viscosity measurements with micro capillary dies are indispensable for the development of microinjection techniques. Precise theoretical modeling and experiments were performed by Wu et al. [3]. It was suggested that the reduction in the initial amount of polymer and increasing pre-compaction pressure improve the accuracy of the measurement. On the other hand, Saari et al. revealed that the glass transition temperature and mechanical properties of poly(vinyl alcohol) can be modified by adding magnesium salts [4]. Interestingly, the salts generally enhance the modulus and yield stress while reducing the crystallinity of the polymer.

Polyelectrolytes exhibit characteristic dynamical behaviors. The structure and diffusivity of microgels were investigated by using plasma etching, which is a unique technique that is useful for clarifying the density distribution within the gel particles [5]. The diffusion of solvent through the particles was also studied in conjunction with the density distribution. The phase separation behaviors of unique star-shaped polyelectrolytes were investigated via turbidimetry [6]. The effect of the structure of the salt and polymer on the phase separation temperature was revealed and the experimental data of the dynamics of the phase separation process was also reported. Polyampholytes (PA) that have both positive and negative residues along the chain exhibit characteristic behaviors of translocation. Kim et al. investigated the translocation process of random PA chains as a model for synthetic PAs under electric field via Monte Carlo simulations [7].

In nanoconfined polymeric systems, the surface and interface of the material influence significantly the polymer chain dynamics. The polymer/substrate interface in a supported polymer film induces specific interactions which significantly alter the dynamics near the interface, and when the interactions are strong, irreversible adsorption of polymer molecules to the substrate surface takes place. The kinetics of this physisorption is not well understood. The interfacial interactions including bonded interactions between polymers and silicate glasses was extensively investigated with atomistic modeling, as described in a review article in this Special Issue [8]. The influences of surface morphology and ambient humidity were also demonstrated. Ishihara et al. investigated the physisorption process via chip nano-calorimetry with temperature modulation for the first time [9]. Their results suggested that the adsorption process is affected by bulky side groups [9].

The specific structure near the interface must be elucidated in order to discuss the interfacial phenomena of polymers. Schlebrowski et al. investigated the structure of the interface between polyamide and amorphous carbon [10]. The samples were prepared via a plasma-supported deposition method under various conditions, and the interlayer that exists at the interface was revealed to be decisive for the adhesion at the interface. In another intriguing study on the dynamical feature near the interface, Hollingsworth et al. demonstrated the hysteretic swelling dynamics of polyelectrolyte brushes deposited on a substrate [11]. Such a study provides useful findings on the fabrication of functional surfaces and interfaces.

Crystallization process of polymers includes the rearrangement of polymer chains, and thus it is one of the interesting topics in the field of polymer dynamics under a non-equilibrium condition. Ramos et al. investigated the crystallization of linear chains under one-dimensional confinement via Monte Carlo simulations [12]. The influence of the hard wall of confinement on the crystallization process and the structure of the resulting crystals were elucidated.

It should be noted that the present issue includes two contributions that concern polymer synthesis, where the molecular dynamics affect the polymerization process. Galukhin et al. studied the kinetics of polymerization of a novel tricyanate ester [13]. They observed a transition from a kinetic- to a diffusion-controlled regime for the polymerization process. Hoffmann et al. prepared hydroxy-terminated polyoxymethylene-*co*-polyoxyalkylene multi-block telechels to produce high-performance thermoplastics [14]. The article includes experimental data that are related to the reaction kinetics.

As described above, the articles in this Special Issue report intriguing topics of polymer dynamics in a wide variety of characteristic systems. At the end of this Editorial, it is worth stressing that further effort is required to achieve a deeper microscopic understanding of the dynamical phenomena in supercooled liquids and glasses, although quite a few theoretical approaches have been developed so far. In particular, the mechanism of the viscous slowing down of relaxation at low temperatures has not yet been fully elucidated. In conjunction with this viscous slowing, the occurrence of a thermodynamic phase (ideal glass) transition is still in dispute. This is one of the most challenging subjects in condensed matter physics.

Furthermore, further attempts should be made to explain the mechanism of the anomalous dynamics in nanoconfined polymeric systems. It is likely that the scaling relation between the cooperativity and the energy barrier for the segmental rearrangement depends on the degree of confinement, and that this is the cause of the different dynamics in polymer ultrathin films compared to those in the bulk. Indeed, the size dependence of the glass transition temperature observed from a pseudo-thermodynamic technique such as volumetry does not coincide with that of a dynamical technique such as dielectric spectroscopy [15]. One may infer that the distribution of segmental relaxation is drastically broadened in nanoconfined systems compared to that of a bulk system due to the effects of finite-size and surface/interface, and the broadened distribution of relaxation may give rise to the apparent anomality in the confined systems.

In addition, the origin of the dynamical heterogeneity observed in supercooled liquids is another crucial issue in this research field [16]. This phenomenon might be originated purely from the dynamical feature, and it may not be simply connected to some spatial heterogeneity in the structure such as density fluctuations. We have demonstrated that rather broad distributions of cooperativity can be derived by just assuming the fluctuations in energy [1]. Further investigations are expected to be performed on this subject.
